# Elastic Laminar Reorganization Occurs with Outward Diameter Expansion during Collateral Artery Growth and Requires Lysyl Oxidase for Stabilization

**DOI:** 10.3390/cells11010007

**Published:** 2021-12-21

**Authors:** Ryan M. McEnaney, Dylan D. McCreary, Nolan O. Skirtich, Elizabeth A. Andraska, Ulka Sachdev, Edith Tzeng

**Affiliations:** 1 VA Pittsburgh Healthcare System, Pittsburgh, PA 15240, USA; ddm27@pitt.edu (D.D.M.); nos36@pitt.edu (N.O.S.); tzenge@upmc.edu (E.T.); 2 Division of Vascular Surgery, University of Pittsburgh School of Medicine, Pittsburgh, PA 15213, USA; andraskaea@upmc.edu (E.A.A.); sachdevu2@upmc.edu (U.S.)

**Keywords:** arteriogenesis, arterial structure, extracellular matrix, peripheral arterial disease, collateral circulation

## Abstract

When a large artery becomes occluded, hemodynamic changes stimulate remodeling of arterial networks to form collateral arteries in a process termed arteriogenesis. However, the structural changes necessary for collateral remodeling have not been defined. We hypothesize that deconstruction of the extracellular matrix is essential to remodel smaller arteries into effective collaterals. Using multiphoton microscopy, we analyzed collagen and elastin structure in maturing collateral arteries isolated from ischemic rat hindlimbs. Collateral arteries harvested at different timepoints showed progressive diameter expansion associated with striking rearrangement of internal elastic lamina (IEL) into a loose fibrous mesh, a pattern persisting at 8 weeks. Despite a 2.5-fold increase in luminal diameter, total elastin content remained unchanged in collaterals compared with control arteries. Among the collateral midzones, baseline elastic fiber content was low. Outward remodeling of these vessels with a 10–20 fold diameter increase was associated with fractures of the elastic fibers and evidence of increased wall tension, as demonstrated by the straightening of the adventitial collagen. Inhibition of lysyl oxidase (LOX) function with β-aminopropionitrile resulted in severe fragmentation or complete loss of continuity of the IEL in developing collaterals. Collateral artery development is associated with permanent redistribution of existing elastic fibers to accommodate diameter growth. We found no evidence of new elastic fiber formation. Stabilization of the arterial wall during outward remodeling is necessary and dependent on LOX activity.

## 1. Introduction

Collateral artery growth is a spontaneous, life- and limb-preserving response to arterial occlusive disease (AOD) that maintains end-organ perfusion when a larger conductance artery becomes occluded. The current consensus suggests collateral arteries develop from pre-existing interconnecting arterioles that bridge separate arterial territories [1]. Many collaterals will originate as small nutrient vessels [2]. With obstruction of a conductance artery (as occurs in peripheral arterial disease), flow pattern changes initially elicit vasodilation in these small vessels. When flow changes are sustained, inflammatory cell recruitment and structural expansion occurs. Ultimately, these transformed vessels become clinically important conduits that maintain target tissue perfusion [3]. This process has been termed “arteriogenesis” and has greater capacity for restoring tissue perfusion than angiogenesis or vasculogenesis, under such circumstances [4,5].

While the signaling mechanisms governing arteriogenesis remain incompletely defined, many key features have been described. Mechanotransduction of increased fluid shear stress leads to endothelial activation in the early stages. Inflammatory cell recruitment and cytokine production precede structural remodeling, which may be increased with local oxidative stress [6,7,8]. Resident cells of the arterial wall proliferate, expanding the mass by up to 50-fold [9]. Proteases including matrix metalloproteinases are upregulated, facilitating luminal enlargement by releasing the elastic and collagen fiber constraints within the arterial wall [10,11]. Histologically, the internal elastic lamina (IEL) has been described as becoming fragmented and transiently disappearing in vessels undergoing arteriogenesis [12]. While it is intuitive that the extracellular matrix (ECM) must be altered to permit arterial remodeling and expansion, the nature of this restructuring has not been characterized and is the focus of our study.

A comprehensive analysis of the three-dimensional microstructure of the arterial wall has led to insights into structural diseases such as Marfan syndrome and aortic dissection [13,14,15]. Multiphoton microscopy (MPM) is very effective for a high-resolution three-dimensional analysis of matrix components within intact arterial tissues [16]. Given the nonlinear optical properties of the arterial ECM, a combination of two-photon excited fluorescence and second-harmonic generation microscopy with spectrally selective detection can be used to image tissues without chemical or immunological staining.

In this study, we used MPM to characterize elastic and collagen structure through arteriogenesis to understand the morphological alterations that permit outward arterial remodeling. These findings will improve our knowledge of arteriogenesis and guide further investigations into the molecular mechanisms of collateral development.

## 2. Materials and Methods

Additional data that support the findings of this study are available from the corresponding author upon reasonable request.

### 2.1. Animal Models and Tissue Harvest

All animal procedures were approved by the Institutional Animal Care and Use Committee at the University of Pittsburgh (IACUC Protocol #19095696) and were performed in accordance with the NIH Guide for the Care and Use of Laboratory Animals and ARRIVE guidelines. A total of 86 male Sprague Dawley rats (8–12 weeks old, Envigo RMS) were used over the course of the study. Rats were anesthetized with intraperitoneal ketamine (75–100 mg/kg, Covetrus) and xylazine (10 mg/kg, Akorn) prior to any surgical or euthanasia procedure. Buprenorphine (0.1 mg/kg, Covetrus) was administered subcutaneously for analgesia, every 12 h following surgical procedures for a total of 48 h. All efforts were made to minimize animal suffering.

**Figure 1:**
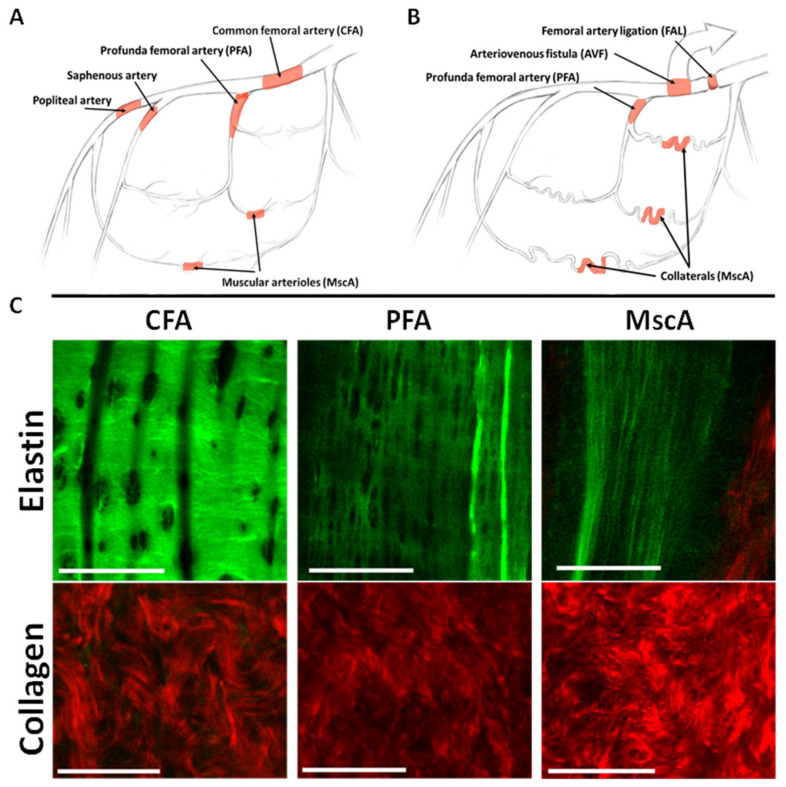
Baseline extracellular matrix structure in various arteries. Shown is a simplified schematic illustration of the lower limb arterial tree in rodents (**A**) and after placement of FAL + AVF in our model (**B**). (**C**) Representative multiphoton microscopy images taken from the intima and adventitia demonstrate the IEL elastin (false colored green) and adventitial collagen (false colored red) of baseline arterial structures. Bar = 50 µm. N = 3 vessels imaged per group. Images acquired with Olympus FV1000MPE utilizing 830 nm laser.

To stimulate arteriogenesis, a modified model of hind limb ischemia was performed as previously described [17]. Briefly, rats were given 50–70 U of heparin (Sigma-Aldrich) in 0.9% saline intravenously. Femoral artery ligation (FAL) was performed at the proximal common femoral artery (CFA), and an arteriovenous fistula (AVF) was created between the distal CFA and common femoral vein (CFV, Illustrated in Figure 1A,B). The FAL + AVF increases flow across the arteriolar connections compared to FAL alone, thereby stimulating growth of collateral networks with greater size and conductance [17,18]. In a subset of animals, the AVF was re-exposed 4 weeks later and closed with a ligature at the distal CFA. Sham operation consisted only of a surgical exposure of the CFA and CFV. To evaluate the role of lysyl oxidase (LOX) in arterial remodeling, rats were treated with the irreversible LOX inhibitor β-aminopropionitrile [19] (BAPN, 0.02% *w*/*v*, ACROS Organics) in drinking water starting 48 h prior to FAL + AVF for up to 8 weeks. Laser speckle contrast imaging (LSCI) was performed on anesthetized animals with the region of interest defined as the plantar paw (Moor FLPI-2, Moor Instruments, Inc.; Wilmington, DE, USA). Animals were euthanized under anesthesia (as above) at different time points via cardiac puncture and exsanguination.

For intraluminal elastase exposure, the CFA was temporarily occluded proximal and distal to the superficial epigastric artery (SEA) branch. The SEA was cannulated with a microrenathane catheter (0.025″ diameter, Braintree Scientific; Braintreee, MA). Porcine pancreatic elastase (PPE, 4–8 U/mL, 100–200 µL, Sigma-Aldrich; St. Louis, MO, USA) solution was instilled into the CFA and PFA branch through the catheter and allowed to dwell for 5 min prior to evacuation and irrigation with saline. The catheter was removed, and the SEA was ligated. Flow was then restored into the CFA.

Upon euthanizing animals, the aorta was then catheterized and flushed with warm heparinized saline followed by 2% paraformaldehyde. The lower limb arterial tree was dissected, and the CFA, profunda femoral (PFA), and iliac arteries were resected en bloc. For microscopy, approximately 1 mm arterial segments were opened longitudinally for en face imaging. Some vessels were prepared for frozen sectioning and confocal imaging (Olympus FluoView 1000) or MPM (Olympus FV1000MPE and 830 nm laser). Immunostaining was performed with anti-LOX (ab174316, AbCam; Waltham, MA, USA) and anti-α-smooth muscle actin (PA5-18292, Invitrogen; Carlsbad, CA, USA).

### 2.2. Human Arterial Tissue

Human arterial specimens were isolated from discarded lower extremity surgical amputations performed for clinically advanced ischemic disease. Informed consent for retention and analysis of deidentified discarded clinical specimens and clinical images was obtained by the clinical physician team. Arterial tissues were harvested in accordance with the Declaration of Helsinki principles and the University of Pittsburgh institutional guidelines (IRB Protocol #PRO11070041). When a CT scan or arteriogram was available, it was used as reference to facilitate location and characterization of collateral arteries. Collateral specimens were collected from both trans-tibial and trans-femoral amputations and originated as muscular arterioles or peri-neural feeding vessels. These vessels were fixed in 2% paraformaldehyde for microscopy.

### 2.3. Multiphoton Microscopy

For whole-vessel images, arteries were opened longitudinally and placed en face in drop slides submerged in PBS. Simultaneous two-photon excitation and second-harmonic generation were used to image the elastin and collagen, respectively. The RXD1 channel (350–450 nm emission filter) allowed visualization of the fibrillar collagen via second harmonic generation, while the RXD2 channel (500–550 nm emission filter) was used to image the elastic fiber autofluorescence via two-photon excitation. Image stacks were acquired at 1024 × 1024 pixels (508.4 × 508.4 µm) per frame with a 1 µm step-size at 8–10 µs per pixel exposure time. For whole vessels, with a 3× line Kalman filter was applied, which uses a recursive algorithm to integrate multiple sweeps of the laser and reduce stochastic noise. For en face images and cross sections, a 4× line Kalman integration filter was applied.

### 2.4. Isolation of Desmosine from Blood and Tissue

Blood was collected from the CFV at the initial and final procedures; it was allowed to coagulate at room temperature and was then centrifuged. Serum was collected and stored at −80 °C for further analysis. Arteries were flushed with heparinized saline through an aortic cannula, and arterial tissues were harvested and stored in cold PBS. Arterial tissue samples were hydrolyzed in 6N HCl heated to 106 °C for 48 h. These samples were subsequently desiccated using a SpeedVac evaporator (ThermoFischer; Waltham, MA, USA) to remove acid and then resuspended in water for analysis. The desmosine content of acid hydrolyzed tissues or serum was quantified using ELISA following manufacturer’s instructions (MyBiosource; San Diego, CA, USA).

### 2.5. Morphometric Analysis of Internal Elastic Lamina Fenestrae and Adventitial Collagens

Analysis of IEL fenestrae was performed using Nikon NIS Elements (version 5.21.01, Nikon Inc.; Belmont, CA). Image stacks for each vessel analyzed were cropped in *z* to the 6–12 frames depicting the internal elastic lamina (IEL), then in *xy* to exclude darker image periphery, leaving the central 150,000 to 200,000 µm^2^ space. Shading correction and background subtraction were performed as necessary. A binary threshold was defined to select fenestrations. Representative ROIs were then drawn to select most of the image area, excluding low-contrast spaces, visible wrinkles, or other imaging artifacts.

Second harmonic generation was used to image the adventitial collagen structures. Maximum intensity projections using image stacks of the adventitial collagen were produced, then fibers were traced (path length, L). Straight lines from end to end of the tracing were drawn to give the point-to-point distance (D). Finally, a ratio was taken of the distance to the path length, to give an undulation index, UI (UI = D/L), as reported previously [20]. The undulation index can be used to quantify the waviness of fibers, with a maximum possible UI of 1.0 indicating a perfectly straight fiber. Twelve to fifteen fibers were used to get an average UI from each image, and 4–7 animals at 1–2 images (258,501 µm^2^ frame area) per animal were used to get an average undulation value for a given vessel type. Collagen fibers were traced from en face images where possible but were obtained from whole-vessel preparations of MscA.

### 2.6. Microfil Casting of Arterial Networks

Microfil (MV120-blue; Flow Tech Inc.; Boulder, CO) was prepared per manufacturer’s instructions and infused through the abdominal aorta to create arterial casts. Microfil casts provide improved visualization of collateral vessels during dissection or contrast for micro-CT. Microfil casts were cured at 4 °C for 24 h. The hind limb was dissected to expose superior thigh and pelvic/adductor collateral stems. Images are obtained using an operating microscope fitted with a digital camera (AmScope; Irvine, CA).

### 2.7. mRNA Harvest and qPCR

The profunda femoris artery (PFA) was utilized for evaluation of collateral gene expression. At time of sacrifice, the artery was flushed with cold PBS by placing a catheter in the distal aorta. The PFA was then harvested from the tissue and snap-frozen in liquid nitrogen and stored at −80 °C until use. Frozen tissues were later crushed and further disrupted using Qiagen’s QIAshredder and RNeasy kits to collect mRNA. Reverse transcriptase reactions were performed using Takara’s EcoDry Premix (Oligo dT) kits. Real-time PCR was carried out on Applied Biosystems QuantStudio 6 and 7 Real-Time PCR Systems, using their recommended reagents, plates, and protocols. PCR runs were cycled between 95C and 60C 40 times. Primer sequences were for lysyl oxidase (LOX: 5′–3′ CTGCTGCTGCGTGACAAC; 3′–5′ GACGGCGAGAAACCAACT) and GAPDH (5′–3′ AACCCATCACCATCTTCCAG; 3′–5′ CCAGTAGACTCCACGACATAC). Relative gene expression was calculated using the ΔΔCq method. Expression of target genes was normalized to GAPDH mRNA level. All primers were pre-designed and validated primers for rats specifically and obtained from Integrated DNA Technologies (IDT; Coralville, IA). 4–6 animals were used for each time point.

### 2.8. Statistical Analysis

Results are expressed as mean ± standard error of the mean (SEM). SEMs are represented in graphs as error bars. Four to seven animals were analyzed for each vessel classification. Collagen tissue UIs were analyzed via one-way ANOVA with individual comparisons via Tukey’s HSD with *p*-value correction for multiple comparisons. Comparisons are performed using an independent *t*-test or one-way ANOVA with Tukey’s multiple comparison test unless otherwise indicated. *p*-values <0.05 are considered statistically significant. Statistical details for specific experiments, including *n* values, can be found in figure legends. All calculations were performed in GraphPad Prism 8 statistical software suite.

## 3. Results

### 3.1. Baseline IEL and Collagen Morphology and Composition in Lower Limb Arterial Tree

To evaluate the structure of the ECM at different locations of the normal arterial tree, vessels were isolated from the regions illustrated in Figure 1A. A schematic example of a collateral network occurring in our model is shown in Figure 1B. According to Longland’s classification [21] of collateral vessels, branches of the internal iliac artery represent collateral stems, the muscular vessels and arterioles (MscA) become the collateral midzone, and the profunda femoral artery (PFA) is the re-entry component. MPM images of arterial elastin and collagen at baseline are shown in Figure 1C. The IEL of large elastic arteries such as the CFA appears as a thick, wrinkled sheet of elastin containing numerous small and rounded fenestrations. In primary branches such as the PFA, the IEL maintained a wrinkled topography but was distinguished by more elongated fenestrations. Lastly, the more distal nutrient vessels (MscA) exhibit a fine meshwork of elastic fibers in place of a sheet-like IEL. The adventitial collagen fibers had a similar wavy configuration in all vessels that were imaged.

### 3.2. Structural Remodeling of IEL during Arteriogenesis

**Figure 2:**
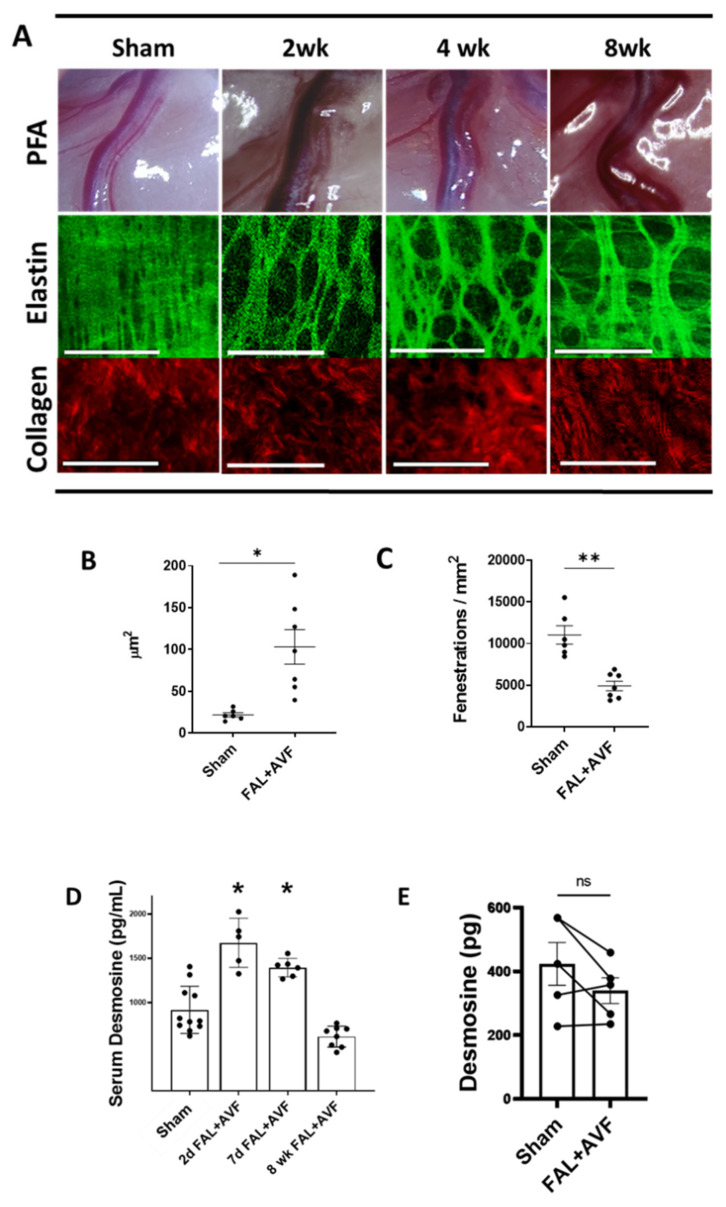
Structural remodeling of the ECM in arteriogenesis. (**A**) Gross and multiphoton microscopic images of PFAs isolated from rats undergoing FAL + AVF or sham operation at various time points. IEL characteristics of fenestration size (**B**) and fenestration density (**C**) were quantified from image stacks for FAL + AVF PFAs (*n* = 7 vessels imaged, range 4–8 weeks post-FAL + AVF) and compared to the contralateral sham PFAs (*n* = 6 vessels imaged). Analysis by two-tailed *t*-test. * *p* < 0.005, ** *p* = 0.003, compared to sham PFA. Bar = 50 µm. Images acquired with Olympus FV1000MPE utilizing 830 nm laser. (**D**) Serum obtained from FAL + AVF animals at indicated time points was measured for desmosine content by ELISA and compared with serum obtained after sham operation. Analysis by one-way ANOVA with Tukey’s multiple comparison test (*n* = 5–11 animals, * *p* ≤ 0.001 compared with control). (**E**) Isolated PFAs underwent acid hydrolysis and desmosine content was quantified via ELISA, *n* = 5 vessels (*p* = 0.13, two-tailed paired *t*-test).

To examine the temporal changes in the arterial wall during arterial wall expansion, we analyzed PFAs removed at 2, 4, and 8 weeks following FAL + AVF (Figure 2A). PFA diameters increased by 255 ± 19% after 4 weeks and became increasingly tortuous. As early as 2 weeks, the IEL had transformed into a loose-appearing meshwork of elastic fibers, a pattern persisting at the 8-week time point. In contrast, adventitial collagen was morphologically unchanged in FAL + AVF PFAs compared to PFAs from sham-operated limbs. The collagen maintained a relaxed and wavy, ribbon-like appearance despite the 2.5-fold vessel diameter enlargement. While the reorganization of the IEL appeared variable, the general pattern consisted of enlarged fenestrations bordered by cords of branching elastic fibers. Occasionally, islands of sheet-like intact IEL were seen separated by exaggerated fenestrations. Average fenestration size was increased in FAL + AVF animals when compared to the contralateral sham vessels (Figure 2B). Overall, these changes resulted in fewer fenestrations visible per unit area in PFAs that were exposed to FAL + AVF (Figure 2C).

We evaluated whether proteolytic degradation of the IEL was necessary for the diameter expansion of arteriogenesis. We collected blood from animals following FAL + AVF or sham operation. At days 2 and 7, there was a significant increase in circulating desmosine in the FAL + AVF rats compared to animals undergoing sham operation, a difference that disappeared at 8 weeks (Figure 2D). These findings suggest that tissue elastin degradation occurs early during arteriogenesis. We suspected that during the maturation of the collateral artery over time, the elastic tissue content would increase along with the vessel size. However, total elastin content remained unchanged in PFAs 8 weeks following FAL + AVF when compared to their contralateral sham-operated controls despite the significant structural enlargement of these arteries (Figure 2E).

**Figure 3:**
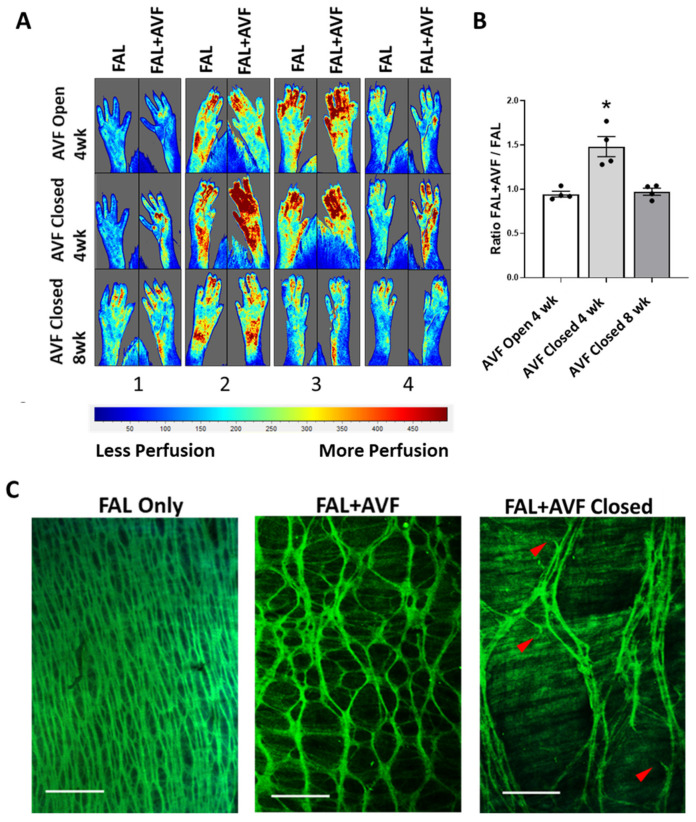
Late closure of the fistula. Subjects initially underwent FAL + AVF with a FAL only on the contralateral hindlimb. After 4 weeks of recovery, animals then underwent a second procedure whereby the arteries were re-exposed. When indicated, FAL + AVF closure was performed by placing a ligature on the distal CFA, preventing any additional direct arterial flow into the venous system. (**A**) Laser scatter contrast images (LSCI) show hindlimbs before and immediately after AVF closure at 4 weeks, then again at 8 weeks. (**B**) Perfusion was essentially normalized between the two limbs at 4 weeks and increased immediately after closure of FAL + AVF (*n* = 4 animals, * denotes *p* = 0.002 when compared to other groups, One-way ANOVA with Tukey’s test), and notably re-normalized to the FAL hindlimb after another 4 weeks. (**C**) Sample projections of IEL from rat PFAs after a total of 8 weeks. Images demonstrate that IEL structure becomes more dispersed with apparent elastic fiber breakage (red arrowheads) and longitudinal consolidation of elastic cords after AVF closure is performed. Image acquired with Olympus FV1000MPE utilizing 830 nm laser. Bar = 50 µm. *n* = 4 vessels imaged per group.

Others have demonstrated that average IEL fenestration size becomes reduced with inward remodeling in models of chronic hypertension [22,23]. Our model, in contrast, lowers circumferential wall strain (by decreasing intraluminal pressure and pulsatility) to the arterial wall in the areas of outward remodeling. We considered that the reduced circumferential mechanical strain to the arterial wall may, in part, precipitate the IEL change. We have previously shown that after collateral development due to FAL + AVF, pressure and pulsatility were increased significantly in the distal femoral artery following AVF closure as compared to FAL alone [17]. To determine whether the alterations in the IEL fenestration structure in the collateral arteries would be reversed removing the AVF from the developed collateral networks, the AVFs were closed 4 weeks after FAL + AVF. In the presence of a functioning AVF, hindlimb perfusion as measured by LSCI had normalized between FAL + AVF and FAL only at 4 weeks, suggesting effective compensation despite AVF presence (Figure 3A,B). Immediately upon closure of the AVF, hindlimb perfusion significantly increased compared to the contralateral FAL-only hindlimb (Figure 3A,B). Four weeks after AVF closure, the PFA showed persistence of the mesh-like IEL pattern without replenishment of elastic tissue or return to baseline structure (Figure 3C). Instead, the IEL underwent further degeneration with appearance of broken elastic fibers and irregular gaps in elastic structure. One PFA demonstrated extensive loss of IEL continuity (Supplementary Figure S1, #4).

### 3.3. Collagen Strain Increases as Function of Fold-Increase over Normal Diameter in Remodeled Inter-Arteriolar Collaterals

**Figure 4:**
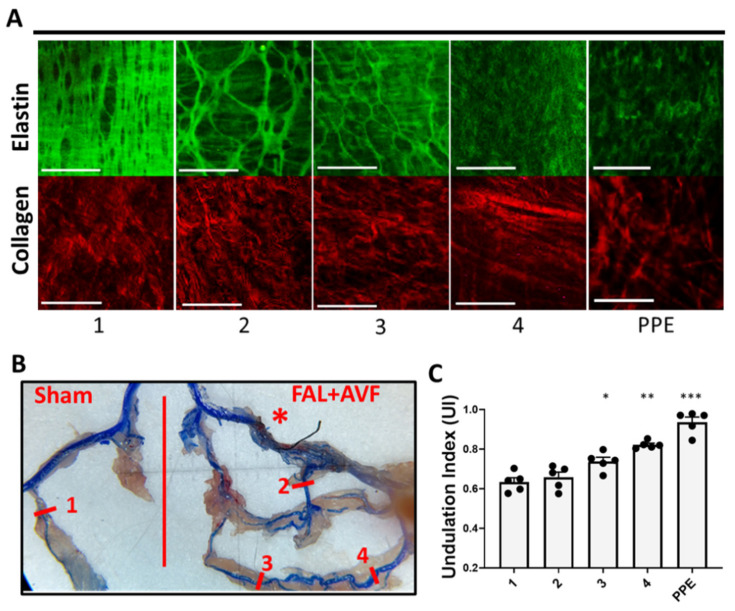
Elastic fiber continuity is stressed with greater relative diameter enlargement. (**A**) Images of the IEL and adventitial collagen isolated from various arterial segments (#1–4) at 4 weeks, compared to elastase-treated common femoral arteries (PPE). Images acquired with Olympus FV1000MPE utilizing 830 nm laser. Sham PFA (1), from the FAL + AVF limb: PFA (2), and sections from MscAs (3,4). (**B**) Microfil infused to the arterial tree to aid in dissection of small muscular collateral vessels. Numbered line segments corresponding to imaging locations of (**A**,**C**). Red * indicates location of FAL + AVF. (**C**) Average adventitial collagen undulation index was calculated for each vessel (comparisons made by one-way ANOVA with Tukey’s multiple comparisons). N = 5 vessels imaged and analyzed per location shown in B. * 3 compared with 1, *p* < 0.05; ** 4 compared with 1 and 2, *p* < 0.001; and *** PPE compared with 1–4, *p* < 0.05. Bar = 50 µm.

The greatest absolute diameter expansion in our model occurred among the inter-arteriolar connections between adjacent arterial territories (representing the “midzone” of developed collaterals per Longland’s description). These were typically located in long muscle beds of the extremity and the peri-neural arterial vasculature. Collateral arteries rising in these locations dilated approximately 10–20-fold from baseline measurements (100–300 µm from 15–20 µm) in our model. This was particularly salient among collaterals that formed on the side of flow reversal (Figure 4(A4,B4)) (flow pattern changes in arteriogenesis are reviewed in ref. [24]). At baseline, these MscAs have an IEL structure composed of fine elastic fibers with lower elastin content morphologically (see Figure 1C). With increasing diameter expansion in response to FAL + AVF, these bundles became strained, forming a net-like pattern with intermittent breaks in the fibers (Figure 4(A3,B3)). With further expansion, however, the IEL structure became more fragmented or lost entirely, resulting in an ECM structure resembling that of the PPE-treated CFAs (Figure 4(A4,B4)). Adventitial collagens appeared progressively straightened moving from the PFA to the MscA (Figure 4C, 2–4), providing evidence of increased load-bearing as elastic structure degenerated [25]. Comparison of UI [26] among these arteries suggested that collagen remains relaxed until the continuity of the IEL is significantly compromised, at which point mechanical loading of collagen increases.

### 3.4. LOX Is Required for Elastic Tissue Stabilization during Outward Remodeling

**Figure 5:**
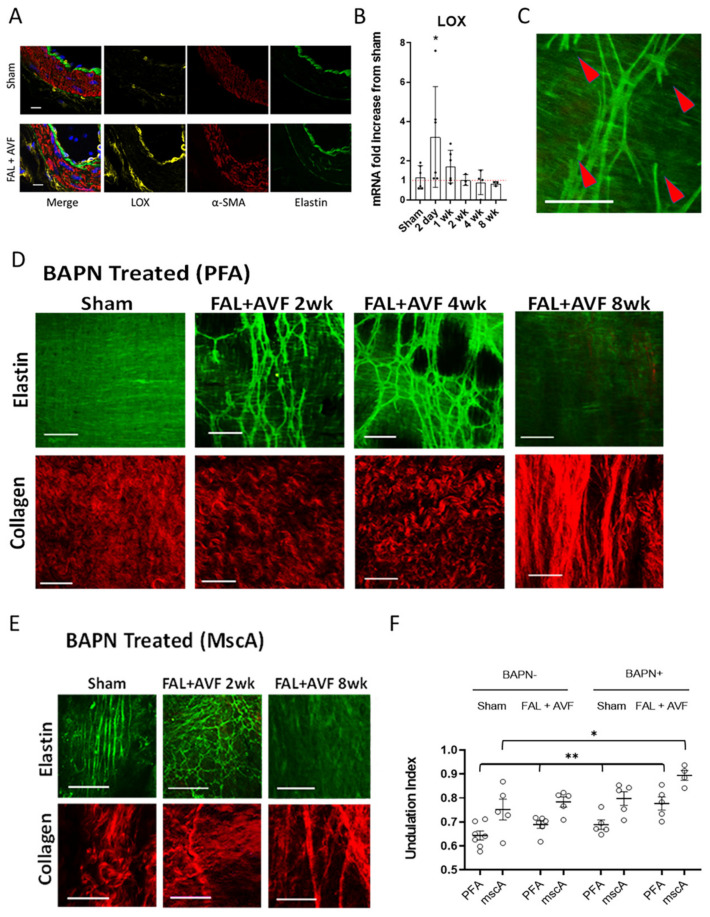
Elastic tissue fragmentation with inhibition of lysyl oxidase. (**A**) Representative cross sections of the PFA at 48 h stained for LOX and imaged with confocal microscopy (bar = 10 µm; *n* = 4). (**B**) LOX mRNA expression is significantly increased in arteries subjected to FAL + AVF relative to sham arteries. (*n* = 3–6, *p* < 0.05) (**C**) Enlarged image of PFA shows stubs of broken elastic fibers appearing in animals treated with BAPN (arrows). PFAs (**D**) and MscAs (**E**) were isolated from animals treated with BAPN after FAL + AVF after various time points, with images showing progressive loss of elastic tissue. (**F**) Comparison of undulation index among collagen fibers of the adventitia at 8 weeks (*n* = 4–7 vessels analyzed per condition). Comparisons by two-way ANOVA, Tukey’s multiple comparison test. * *p* < 0.03, ** *p* < 0.05. Bar = 50 µm. Images acquired with Olympus FV1000MPE utilizing 830 nm laser.

LOX mediates post-translational crosslinking of elastin and collagen residues, which is essential for the structural integrity of the ECM. Given the observations of elastin degradation occurring in early collateral remodeling along with the necessity of maintaining IEL elastic fiber integrity since loss of continuity appears irreversible, we examined the role of LOX during arteriogenesis. LOX protein expression was increased in remodeling PFAs in FAL + AVF animals (Figure 5A) when compared to sham arteries. LOX mRNA was then demonstrated to be significantly upregulated in FAL + AVF animals when compared with sham arteries early in arteriogenesis (Figure 5B). Rats treated with BAPN to inhibit LOX activity showed no change in the elastic fiber structure in sham PFAs, but increased elastin fiber fragmentation was observed in FAL + AVF PFAs (Figure 5C). In 1 of 4 animals at 8 weeks, the IEL continuity was lost entirely, resulting in an ECM morphology that was very similar to PPE-treated arteries (Figure 5D, FAL + AVF 8wk). Collaterals rising from the smaller MscAs were also examined (Figure 5E). MscAs from sham operated limbs had thin, linearly oriented elastic fibers in the IEL. At 2 weeks after FAL + AVF, the MscA IELs in BAPN-fed rats appeared fragmented and disorganized. At 8 weeks, increasingly irregular, enlarged collateral structures were identified that exhibited total loss of elastic fibers (Figure 5E, FAL + AVF 8wk) with straightened collagen bundles (Figure 5F).

### 3.5. ECM Characteristics of Human Collateral Arteries

**Figure 6:**
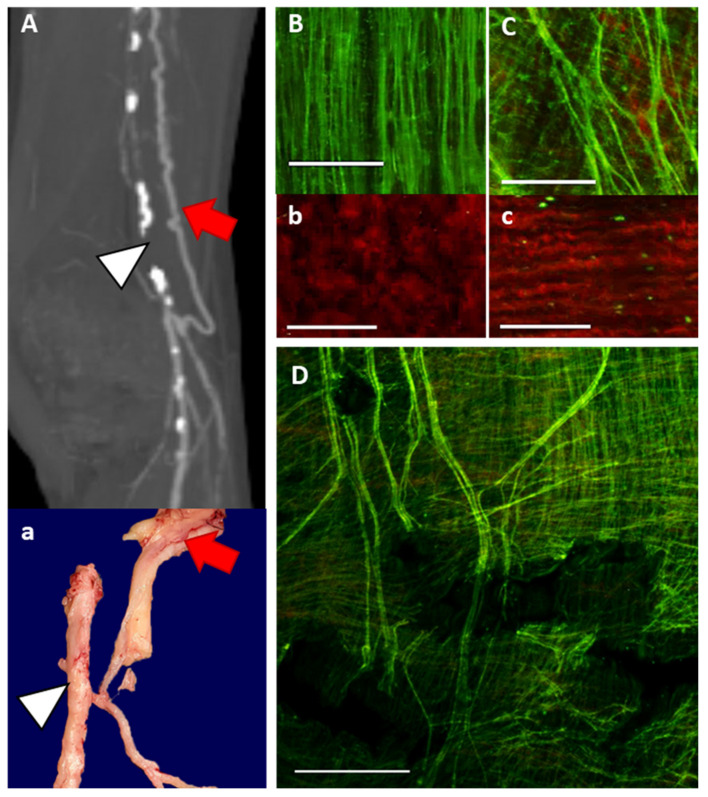
ECM characteristics of functional human collateral arteries. CT arteriography (**A**) and gross specimen (**a**) from patient undergoing trans-femoral amputation for lower limb ischemic gangrene. The occluded femoral-popliteal artery is indicated by white arrowhead, and the collateral artery is shown in red arrow. Notably, the collateral artery is embedded within the sciatic nerve. (**B**,**b**) Baseline elastic and collagen structure from arterial specimen that is not collateralized. (**C**,**c**) Elastic and collagen structure of human collateral artery taken from amputation specimen. Bar = 50 µm. (**D**) Enlarged view of human collateral artery isolated near red arrow (**a**) demonstrating dispersed elastic fibers with numerous breaks, and gaps in elastic substructure. Bar= 100 µm. Elastin appears green and collagen appears red. Images acquired with Olympus FV1000MPE utilizing 830 nm laser.

Collateral arteries isolated from human lower extremity amputation specimens exhibited similar ECM morphology to the collateral remodeling observed in the rat FAL + AVF model. Human collaterals (*n* = 4) were identified within the musculature or closely associated with large nerve structures and were recognizable for their tortuous appearance and thin walls. A preoperative computed tomography arteriogram was available for one such specimen (Figure 6A). The reconstructed imaging clearly identified the occluded superficial femoral and proximal popliteal arteries as well as the large, tortuous collateral artery that reconstituted the popliteal artery at the knee. MPM showed the densely organized elastic structure of the IEL in noncollateralized muscular arteriole (Figure 6B, isolated from a separate specimen), while the elastic fibers in the collateral (Figure 6C) were more dispersed to accommodate diameter growth. Additionally, the corresponding collagen structures were comprised of relaxed, wavy fibers in the noncollateralized arteriole (Figure 6b) but were straighter in the collateral vessel (Figure 6C). A larger-field view of the collateral artery IEL (Figure 6D) demonstrated irregular spacing and fragmentation of the elastic fiber bundles, which are consistent with the changes we observed in the rodent model.

## 4. Discussion

Expanding collateral capacity through arteriogenesis would prove a promising treatment strategy for AOD. Increasing collateral vessel conductance requires greater diameter expansion and greater ECM remodeling of these arterial connections. In this study, we utilized a rodent model of FAL with a downstream AVF to create increased blood flow across collateral arterioles to drive robust arteriogenesis [17]. We observed an apparent 10–20 fold vessel diameter increase among arteriolar interconnections in the collateral midzones, resulting from the flow changes. Diameter expansion occurred with dispersion of existing elastic tissue in the IEL and resultant enlargement of the fenestrations. An important finding of this study was that, despite a considerable increase in diameter, total elastin content of the “collateralized” vessel was not increased when compared to the “noncollateralized” contralateral sham vessel. While we did not observe an increase in elastic fiber content or elastic tissue mass to coincide with vessel diameter increase, it appears that cross-linking of new elastin is of critical importance since inhibition of LOX resulted in rapid fragmentation and total loss of continuity of the remodeling IEL. Taken together, these findings suggest that conservation of existing elastic fibers is necessary for successful arterial remodeling. Further, when IEL continuity is lost in developing collaterals, collagen morphology becomes strained, suggesting that wall tension is transmitted principally to collagen fibers.

The vascular ECM is largely composed of elastic fibers and collagens. The elastic fibers store energy and distribute stress throughout the arterial wall, while the collagen network provides a framework that resists over-distension but is unable to store energy [27]. While the complex interplay between elastin and collagen components of the ECM is necessary for an artery’s structural stability, it also sets limits on vasodilation and must be overcome for collateral artery enlargement to occur [28,29,30,31,32,33].

Elastin biosynthesis occurs predominantly in neonatal and early postnatal life, with negligible new elastin produced in adult tissues [31,34]. Elastin represents 90% of elastic fiber mass, with the remainder composed of peripherally oriented fibrillar proteins and proteoglycans. When elastic fibers are constructed in development, tropoelastin monomers (synthesized by VSMCs and VECs) self-assemble in the extracellular space and associate with the microfibril scaffold where LOX mediates extensive cross-linking modifications [35]. The elastin core appears amorphous in the relaxed fiber but is revealed as a structure of branching filaments with diameters ranging from 1–5 nm when imaged under tension [35,36]. The dense cross-linking of elastin, 4–5 times greater than collagen, underpins both its durability and insolubility. Mature elastin may be the most durable protein in mammals, with an estimated half-life approaching the human life span [37]. In contrast, collagen fibers have short half-lives of the order of two weeks and therefore must undergo continual synthesis for replacement and reinforcement in the arterial wall [38,39].

In axial conduit arteries, elastic fibers coalesce to form thick sheets with fenestrations (referred to as lamellae) in the tunica media. The elastic lamellar superstructure is integrated with VSMCs and filamentous elastic fibers, which are dispersed throughout the media and the adventitia [28,40]. When viewed in cross section under low distending pressures, the numerous elastic lamellae assume a wrinkled or wavy appearance that flattens at higher pressures [27]. The most prominent elastic lamina is the IEL, a unique layer that separates the endothelium from underlying VSMCs. The IEL contains fenestrations that vary dramatically in size, shape and frequency, depending on the location in the arterial tree [28,40,41]. In larger conduit arteries, the IEL is a thick sheet with punctate fenestrations while the branch arteries to skeletal muscle possess a thinner IEL with more numerous and larger fenestrations [42]. We also observed along the arterial tree that more distal branches have diminishing elastic fiber content down to the arteriolar level. A similar pattern was previously reported in the aorta, where elastic content decreases with decreasing lamellae along the linear path of the aorta at arterial branch points [43].

Fenestrations in the IEL are functionally important, facilitating communication between cells residing in neighboring lamellae [41]. They also become active sites of elastic laminar remodeling during postnatal growth of arterial structures [40,44]. In mature arterial tissues, enlarging fenestrations allow for adaptive expansion of IEL surface in models of increased arterial flow and axial tension [45,46]. Conversely, IEL fenestrations contract with inward arterial remodeling in hypertensive rats [23]. IEL degradation has been demonstrated in maturing vein grafts in response to increased fluid shear stress [47]. We similarly observed that structural growth in arteriogenesis is associated with active remodeling at fenestrations, resulting in a dispersed, weblike IEL among large collaterals. In this way, changes in IEL fenestration size may reflect either outward or inward arterial wall remodeling.

Elastic fiber assembly is highly complex and only occurs in development, making vertebrates susceptible to age and disease related degeneration of elastic fibers [34,37,48]. Evidence suggests that damaged elastic fibers can be salvaged if the fiber integrity is preserved [49,50,51]. However, if fiber integrity is lost, disordered elastin synthesis may occur without restoration of functional elastic fibers [52,53]. Our results suggest arterial diameter expansion is followed by stabilization of the reformed elastic tissue, but this does not appear to include creation of new elastic fibers to fill in the voids of larger fenestrations. Notably, these enlarged fenestrations did not remodel again over time in this model nor did elastic fiber bundles return to a more densely populated configuration after closure of the AVF. Therefore, successful outward remodeling requires the reorganization of existing elastic fibers (permitting luminal expansion) while minimizing loss of continuity and strength.

Elastic structural remodeling during arteriogenesis requires elastolytic activity. Important proteolytic enzymes with elastolytic capability that may be produced in the arterial wall include MMPs and cathepsins, which can be upregulated during wall remodeling or inflammation [54,55]. Indeed, proteolytic degradation by matrix degrading enzymes in early arteriogenesis has been reported [11,46,56,57,58]. However, elastolysis necessarily damages the elastic fibers to permit the expansion. Elastolytic activity must be kept in check and damage to elastic fibers repaired to maintain fiber integrity.

While the larger PFA possesses sufficient elastic tissue to allow dispersion of fibers while maintaining continuity during outward remodeling, smaller arterioles have substantially less elastic tissue [59]. We found that while MscAs have the sparsest elastic fiber content at baseline, they undergo the greatest relative diameter growth (10–20 fold) in response to FAL + AVF. The elastic fibers in collateral midzones rising from these arteries appeared strained with hexagonally shaped fenestrations and exhibited occasional breaks or were lost entirely. The fracturing and loss of the elastic fibers was associated with mechanical loading of adventitial collagen. Morphologically, these vessels become similar to experimentally produced aneurysms. As with arterial aneurysms, the loss of elastic fiber continuity appears to be irreversible, and a reappearance of elastic fiber networks was not observed.

During arterial remodeling, LOX serves a critical role in the repair and stabilization of proteolytically damaged elastic fibers. We showed that LOX inhibition did not prevent collateral vessel diameter expansion. However, in remodeling collaterals, LOX inhibition was associated with elastic fiber fractures and loss of IEL integrity, which again morphologically resembles experimental aneurysmal degeneration. These findings suggest that the proteolytic balance likely shifts toward repair with newly synthesized tropoelastin monomers that require cross-linking in the later phases of arteriogenesis. Resident vascular cells likely participate in repair by synthesizing tropoelastin that is incorporated into damaged fibers and expression of LOX to create stabilizing crosslinks among soluble and existing insoluble elastin [49,50,51,60]. Increased tropoelastin expression has been previously demonstrated in models of vascular remodeling [61]. Others have suggested that repair of damaged elastin polymers is unlikely to reestablish the original strength as the original peptide bonds cannot be recovered [50]. Total elastin content fails to keep pace with the increasing vessel size, and the thinly dispersed elastic fiber ultrastructure seen in this study likely also contributes to a weaker IEL than the baseline configuration. Our findings of IEL fragmentation after closure of the AVF (Figure 3, Supplementary Figure S1) suggest that this altered structure, while certainly permitting for diameter expansion and improved flow conductance, is not optimal for cyclic strain and is likely vulnerable to mechanical failure. This, theoretically, could lead to aneurysmal degeneration among vessels undergoing outward remodeling [62]. Additional investigations into the mechanical integrity of the remodeled arterial wall would be informative.

Our analysis of collateral artery specimens harvested from amputated limbs (Figure 6) confirmed that the collateral artery structure modeled in the rat is fundamentally similar to human collateral arteries in the setting of peripheral arteries disease. Additionally, the collateral arteries examined from clinical specimens had likely been present for many years, further supporting the notion that new elastic fibers are not reconstructed. The sparse, fragmented IEL structure in these collaterals further supports the notion that IEL remodeling relies on reorganization and repair of existing elastic fibers but not on new fiber synthesis.

Our study has notable limitations that should be considered. Our model uses an AVF to increase the hemodynamic drive for outward remodeling, resulting in greater network conductance at the cost of introducing artificial hemodynamic complexity. The AVF increases venous pressure of the affected limb, which may have some influence over arteriogenesis and would not be replicated clinical situations. Additionally, it has been long proposed that as collateral networks mature, some select vessels expand and some regress [21]. Our experimental closure of AVFs after 4 weeks did not produce any evidence of “regression” of any collateral arteries to smaller or more normalized ECM architecture in our model. It is possible that the time point we studied was insufficient to demonstrate this. Future work will focus on further understanding collateral network maturation and local expression of genes regulating ECM remodeling, such as collagens, tropoelastin, and elastolytic MMPs and cathepsins. Lastly, this study is primarily descriptive regarding structural remodeling in arteriogenesis but provides important details about the events of arteriogenesis that will serve as the basis for future mechanistic studies.

## 5. Conclusions

Arteries rely on elastic fibers and collagen bundles for structural stability under hemodynamic stress, but these components also create limitations on outward remodeling. The arterial wall ECM is differentially composed along the arterial tree according to vessel size. During arteriogenesis, these matrix structures undergo significant remodeling to accommodate diameter expansion. Existing elastic fibers are partially degraded, allowing for thinning and spreading of the IEL. New elastic tissue does not appear to be created during arteriogenesis. If continuity of the elastic framework remains intact with the help of LOX, the load-bearing capability of the IEL is maintained albeit seemingly weakened. If the elastic framework loses continuity, however, mechanical loading of collagen is evident.

Our study suggests that diameter expansion risks exhausting the local baseline elastic structure (especially in collateral midzones), which could create weakened collateral vessels that lose elastic tissue integrity. Through a more detailed understanding of arterial remodeling mechanisms, a molecular method of expanding collateral networks to improve target tissue perfusion may become possible. In addition, developing mechanisms to improve elastic fiber repair or stimulate de novo elastic fiber creation would conceivably improve collateral artery stability and enhance function. Future studies will be aimed at determining molecular mechanisms of the elastic deconstruction and reinforcement, as well as the durability of remodeled arterial structure, which may indicate viable targets to influence arterial remodeling.

## Data Availability

The data that were generated and/or analyzed during the current study are available from the corresponding author upon reasonable request.

## Supplementary Materials

The following are available online at https://www.mdpi.com/article/10.3390/cells11010007/s1, Figure S1: Closure of AVF after 4 weeks.
