# Supplementary material for: Elastic Laminar Reorganization Occurs with Outward Diameter Expansion during Collateral Artery Growth and Requires Lysyl Oxidase for Stabilization
Source: Cells. 2021 Dec 21;11(1):7. doi: 10.3390/cells11010007 (PMC8750335; doi:10.3390/cells11010007)
Supplement: Supplementary file 1 [file cells-11-00007-s001.zip › cells-1486736-supplementary.pdf]

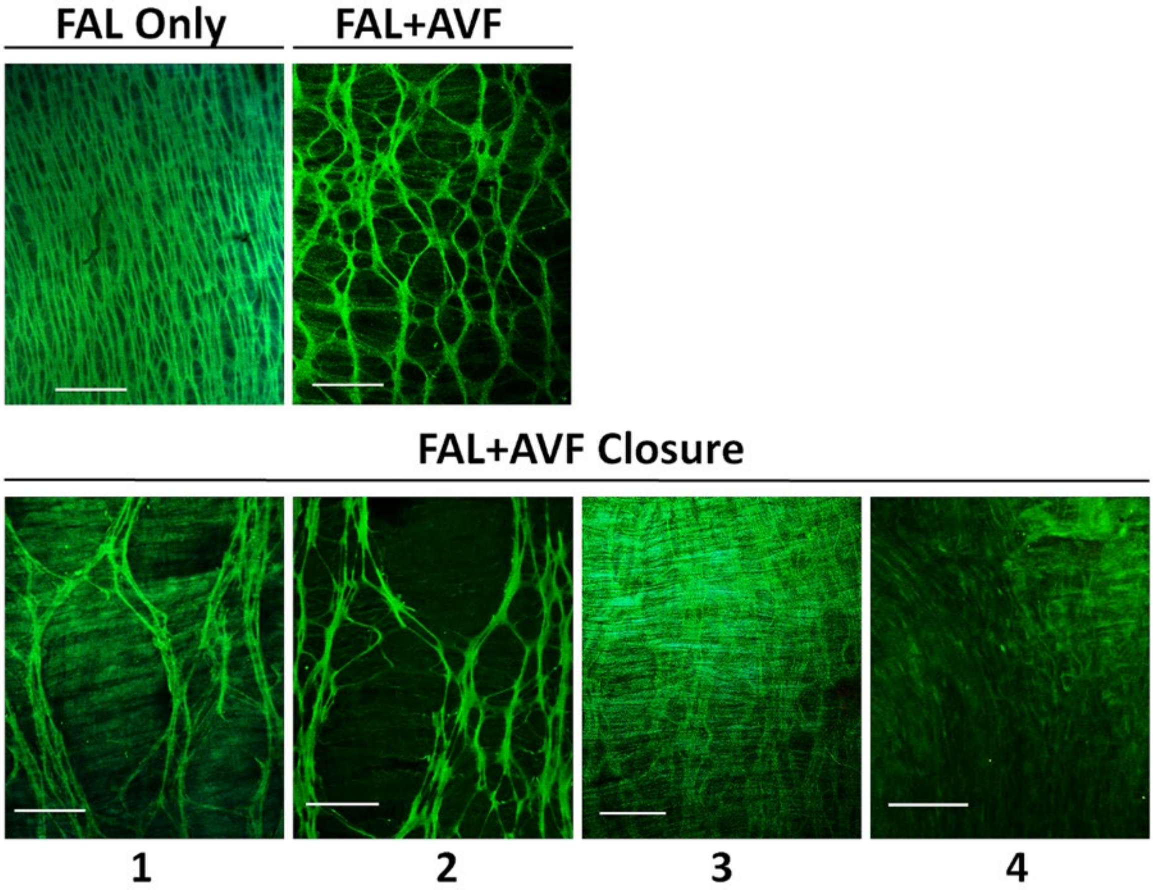

Supplemental Figure SI. Closure of AVF after 4 weeks. PFAs #1-3 demonstrated intact IEL structure but altered with irregular increased gap size between elastic fiber bundles and breakages in elastic fibers. For #4 no remaining IEL visible. Bar = 50 µm. Images acquired with Olympus FV1000MPE utilizing 830 nm laser.
